# Oxidative Stress-Mediated Reperfusion Injury: Mechanism and Therapies

**DOI:** 10.1155/2014/373081

**Published:** 2014-04-03

**Authors:** Zhengyuan Xia, Yanfang Chen, Qian Fan, Mengzhou Xue

**Affiliations:** ^1^Department of Anesthesiology, The University of Hong Kong, 102 Pokfulam Road, Hong Kong; ^2^Department of Anesthesiology, The Second Affiliated Hospital of Guangzhou Medical University, Guangzhou, China; ^3^Department of Pharmacology & Toxicology, Boonshoft School of Medicine, Wright State University, 3640 Colonel Glenn Highway, Dayton, OH 45435, USA; ^4^Cardiovascular Department, Guangzhou Institute of Cardiovascular Disease, The Second Affiliated Hospital of Guangzhou Medical University, Guangzhou 510000, China; ^5^Department of Cardiology, Beijing Anzhen Hospital, Capital Medical University, Beijing 100029, China; ^6^Department of Neurology, The First Affiliated Hospital, Henan University, 357 Ximen Street, Kaifeng 470010, China

Ischemia/reperfusion injury (IRI) and organ failure, especially IRI-induced remote and multiple organ failure, contribute significantly to postoperative mortality and morbidity, and reperfusion induced oxidative stress plays a critical role in this pathology. Postoperative mortality risk increases in aged patients and in patients with concomitant diseases like diabetes which itself is associated with increased oxidative stress. Given that people now live longer and are often with concomitant diseases, IRI in these population is more severe and prevention or treatment of IRI will be an increasing important area of intention.

Ischemic heart disease is a major complication of diabetes [1, 2]. Although expeditious percutaneous coronary intervention can restore coronary flow and limit myocardial infarction, reperfusion may also cause cardiac damage—“ischemia/reperfusion injury (IRI)” [3]. Diabetic heart is more vulnerable to IRI [4, 5] but less sensitive to percutaneous coronary intervention and ischemic pre- or postconditioning cardioprotection, and the underlying mechanism remains unclear. In this special issue, Y. Zhao et al. reported that acute hyperglycemia not only exacerbated myocardial IRI but completely abolished the cardioprotective effect of ischemic preconditioning by inhibiting Akt phosphorylation and disrupting signaling pathways downstream of adenosine A_1_ receptor but not adenosine A_1_ receptor activation itself. Interestingly, insulin treatment to normalize blood glucose levels could restore the cardioprotective effects of ischemic preconditioning, despite that insulin failed to counteract the detrimental effect of hyperglycemia. This is an interesting area that deserves further exploration. Further, M. Liu et al. reported that hyperglycemia-induced inhibition of myocardial DJ-1 protein expression may represent one of the major mechanisms why hearts of diabetic subjects are less or not responsive to ischemic postconditioning cardioprotection.

Remote ischemic preconditioning has recently been shown to effectively attenuate myocardial ischemia/reperfusion injury in patients [6, 7], but the underlying mechanisms are incompletely understood. In this special issue, T. Pang et al. performed remote ischemic preconditioning in healthy volunteers and conducted comprehensive proteomic analysis in order to identify the mechanisms. Furthermore, X. Qiao et al. reported that transient acidosis during early reperfusion can mimic the cardioprotective effects of ischemic postconditioning in a rat model of myocardial IRI induced by coronary artery occlusion/reperfusion. These studies, among others, are significant advancement in the study of ischemic pre- and postconditioning cardioprotection.

Reactive oxygen species (ROS) induced vascular endothelial dysfunction plays an important role in the development of IRI in various organs. In this special issue, J. Wang et al. reported that endothelial progenitor cell-derived microvesicles (EPC-MVs) can promote angiogenesis of endothelial cells and attenuate hypoxia/reoxygenation injury in human brain microvascular endothelial cells. Further, S. Ma et al. reported that the application of low frequency pulse magnetic fields can effectively reduce ROS generation and subsequently attenuate myocardial IRI. Their finding that low frequency pulse magnetic fields could protect the myocardium against IRI via regulating ROS generation and nitric oxide/peroxynitrite balance is a very novel finding that may have high potential to serve as a promising strategy for combating cardiac IRI. X. Zhang et al. reported that Resolvin D1 can effectively protect against impairment of endothelial barrier function induced by lipopolysaccharide in human vascular endothelial cells. This finding may be of significant clinical relevance given that during IRI the increased production of proinflammatory cytokines such as tumor necrosis factor-*α* (TNF-*α*) further increases oxidative stress and exacerbate reperfusion injury [8]. The paper by S. Lei et al. in this special issue reported that TNF-*α* stimulation in endothelial cells can induce oxidative stress primarily through protein kinase C (PKC)-*β*_2_ dependent NADPH oxidase activation and reduce vascular endothelial cell viability.

The intravenous anesthetic propofol possesses antioxidant capacity and has been shown to attenuate IRI in patients undergoing cardiac surgery [9] and in animal models of IRI [10]. However, the mechanisms by which propofol confers protective effects against IRI has not been fully elucidated. In this special issue, Z. Chen et al. systematically analyzed the alterations in microRNA expression in human umbilical vein endothelial cells subjected to hypoxia/reoxygenation in the presence or absence of posthypoxic propofol treatment and provided genome-wide profiling of microRNAs assessed using microRNA microarray.

The experimental therapeutic studies regarding the neuroprotective effect and mechanisms of Ginkgolide B on cell injury reported by L. Li et al. and the effect of safflower yellow on spinal cord IRI reported by D. Zhou et al. are all of potential clinical implications.

The review article by C. Nastos et al. reviewed the existing literature regarding the proposed mechanisms of remote organ injury after liver ischemia and reperfusion. This review brings to our attention the important issue of liver IRI-induced remote organ injuries [11, 12].

We hope that the original and review articles presented in this special issue, representing the current advances in the oxidative stress-mediated ischemia-reperfusion injury, with respect to their potential impact in cellular survival pathways and therapeutic strategies, will stimulate further exploration of this important area.

## References

[1] Donahoe SM, Stewart GC, McCabe CH (2007). Diabetes and mortality following acute coronary syndromes. *The Journal of the American Medical Association*.

[2] Boudina S, Abel ED (2007). Diabetic cardiomyopathy revisited. *Circulation*.

[3] Andersen HR, Nielsen TT, Rasmussen K (2003). A comparison of coronary angioplasty with fibrinolytic therapy in acute myocardial infarction. *The New England Journal of Medicine*.

[4] Miki T, Itoh T, Sunaga D, Miura T (2012). Effects of diabetes on myocardial infarct size and cardioprotection by preconditioning and postconditioning. *Cardiovascular Diabetology*.

[5] Li H, Liu Z, Wang J (2013). Susceptibility to myocardial ischemia reperfusion injury at early stage of type 1 diabetes in rats. *Cardiovascular Diabetology*.

[6] Wu Q, Gui P, Wu J (2011). Effect of limb ischemic preconditioning on myocardial injury in patients undergoing mitral valve replacement surgery: a randomized controlled trial. *Circulation Journal*.

[7] Stazi A, Scalone G, Laurito M (2014). Effect of remote ischemic preconditioning on platelet activation and reactivity induced by ablation for atrial fibrillation. *Circulation*.

[8] Yang YY, Lee PC, Huang YT (2014). Involvement of the HIF-1alpha and Wnt/beta-catenin pathways in the protective effects of losartan on fatty liver graft with ischaemia/reperfusion injury. *Clinical Science*.

[9] Xia Z, Huang Z, Ansley DM (2006). Large-dose propofol during cardiopulmonary bypass decreases biochemical markers of myocardial injury in coronary surgery patients: a comparison with isoflurane. *Anesthesia and Analgesia*.

[10] Liu K-X, Rinne T, He W, Wang F, Xia Z (2007). Propofol attenuates intestinal mucosa injury induced by intestinal ischemia-reperfusion in the rat. *Canadian Journal of Anesthesia*.

[11] Zhang A, Chi X, Luo G (2013). Mast cell stabilization alleviates acute lung injury after orthotopic autologous liver transplantation in rats by downregulating inflammation. *PLoS ONE*.

[12] Hei Z-Q, Li X-Y, Shen N, Pang H-Y, Zhou S-L, Guan J-Q (2008). Prognostic values of serum cystatin C and *β*2 microglobulin, urinary *β*2 microglobulin and N-acetyl-*β*-D-glucosaminidase in early acute renal failure after liver transplantation. *Chinese Medical Journal*.

